# A cadaveric study of the morphology of the extensor hallucis longus - a proposal for a new classification

**DOI:** 10.1186/s12891-019-2688-8

**Published:** 2019-07-03

**Authors:** Łukasz Olewnik, Michał Podgórski, Michał Polguj, Kacper Ruzik, Mirosław Topol

**Affiliations:** 1 Department of Normal and Clinical Anatomy, Interfaculty Chair of Anatomy and Histology, Medical University of Lodz, Lodz, Poland; 2 Department of Diagnostic Imaging Lodz, Polish Mother’s Memorial Hospital Research Institute, Lodz, Poland; 3 Department of Angiology, Interfaculty Chair of Anatomy and Histology, Medical University of Lodz, Lodz, Poland

**Keywords:** Anatomical study, Cadaveric study, New classification, Extensor hallucis longus tendon, Extensor hallucis longus muscle

## Abstract

**Background:**

Morphological variations of the EHL concern mainly the accessory tendons and the site of their insertion. The aim of our study is to present a new classification of the EHL.

**Methods:**

Classical anatomical dissection was performed on 104 lower limbs (51 right, 53 left, fixed in 10% formalin solution).

**Results:**

In the cadavers, three types of morphology (insertion and addidtional band) were observed. Type I, the most common type, was characterized by a single tendon that ends as an extensor hood on the dorsal aspect of the base of the distal phalanx of the big toe (57.7%). Type II was characterized by two distal tendons and was subdivided into three subtypes according to (A-29.9%, B-4.8% and C-5.7%). Type III was characterised by three distal tendons (two cases - 1.9%).

**Conclusion:**

The EHL presents high morphological variability. Knowledge of particular types of insertion is essential for both clinicians and anatomists.

**Electronic supplementary material:**

The online version of this article (10.1186/s12891-019-2688-8) contains supplementary material, which is available to authorized users.

## Background

The extensor hallucis longus (EHL) is a thin muscle situated deep between the tibialis anterior muscle (TAM) and the extensor digitorum longus (EDL). The EHL arises from the middle half of the fibula and from the interosseous membrane, medial to the origin of the EDL. The muscle belly becomes a long tendon, passes behind the superior and inferior extensor retinaculum, crosses the anterior tibial artery and vein from the lateral to the medial side near the ankle, and finally inserts on the dorsal aspect of the base of the distal phalanx of the big toe [1]. The function of the EHL is to extend the big toe, dorsiflex the foot, adjunct foot eversion and inversion and stretch the plantar aponeurosis [1, 2].

The EHL is characterized by morphological variability with regard to the number of its additional bands and their insertion [1, 3–7]. Most previous research has focused on its possible morphological variations, particularly those regarding the additional bands. One classification has been proposed for these variations [3]; however, it requires systematization and upgrading to account for the identification of new band types.

The aim of our study is to systematize the classification of EHL insertion and course of its tendons.

## Methods

### Anatomic studies

One hundred and four lower limbs (50 paired, 62 male, 42 female, 51 right and 53 left) were obtained from adult Caucasian cadavers, and fixed in 10% formalin solution before examination. The mean age “at death” of the cadavers was 64.1 years (35–88). The cadavers were the property of the Department following donation to the university anatomy program. Any lower limbs with evidence of surgical intervention in the dissected area were excluded. All dissection of the leg and foot area was performed in accordance with the pre-established protocol [8–12].

#### Description of the dissection protocol

The first step was to remove the skin and superficial fascia of the leg up to the crural fascia. This step was followed by the removal of the skin and subcutaneous tissue from the area of the foot. With the muscles exposed, as much of the crural fascia as possible was removed, beginning with the retinaculum. Care was taken not to damage the muscle bellies. Following this, the muscle bellies and the tendons were cleaned from the medial to lateral side. The clean muscle bellies were then separated from each other into tibialis anterior, extensor digitorum longus and extensor hallucis longus. Finally, the tendon was dissected to allow examination of the courses of the main and accessory tendons.

Upon dissection, the morphological features of the EHL were assesed:

- The types of EHL insertion
- The course of EHL tendon
- Morphometric measurements of the EHL.

An electronic digital calliper was used for all measurements (Mitutoyo Corporation, Kawasaki-shi, Kanagawa, Japan). Each measurement was carried out twice with an accuracy of up to 0.1 mm. Consent for anatomic studies was obtained from the Local Bioethical Commission (agreement no RNN/297/17/KE).

### Statistical analysis

The results are presented as mean and standard deviation unless otherwise stated.

The normality of the continuous data distribution was checked with the Shapiro-Wilk test. As the data was not normally distributed, the Mann-Whitney test and the Wilcoxon sign-rank test were used to compare anthropometric measurements between sexes and body sides, respectively. The Kruskal-Wallis ANOVA with dedicated post hoc test was used to compare these measurements between EHL types. The statistical analysis was performed using Statistica 12 software (StatSoft Polska, Cracow, Poland). A *p*-value lower than 0.05 was considered significant.

## Results

### Anatomic studies

**Fig. 1:**
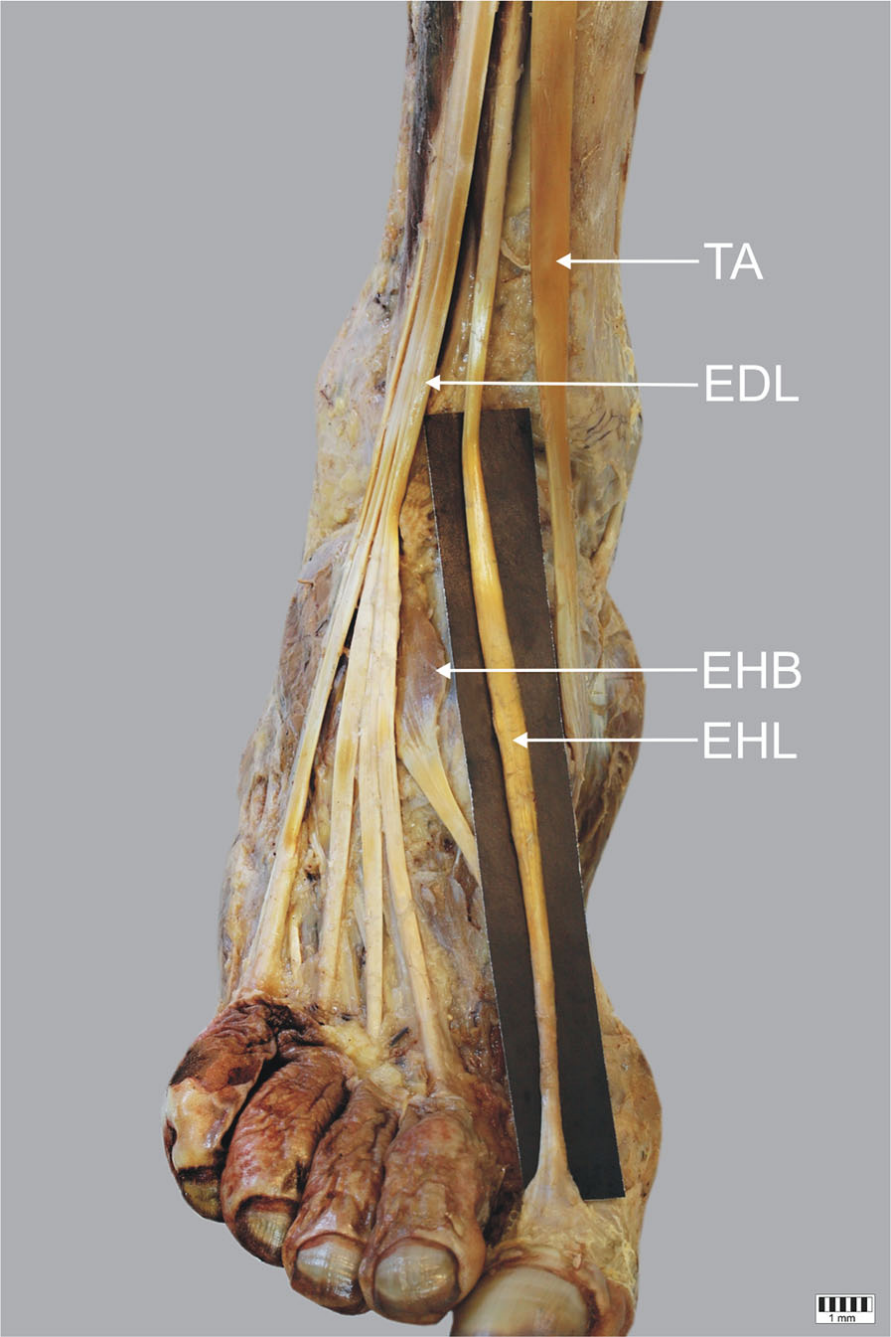
Type I of insertion of the extensor hallucis longus tendon. Right lower limb. *EHL* extensor hallucis longus tendon *EHB* extensor hallucis brevis muscle *EDL* extensor digitorum longus *TA* tibialis anterior tendon

The EHL was present in all specimens. It was classified into three main types based on variation in the morphology of the tendon and its distal attachment:

Type I – a single tendon that ends as an extensor hood inserts on the dorsal aspect of the base of the distal phalanx of the big toe. It was the most common type observed in 60 cases (57.7%) – Fig. 1.

**Fig. 2:**
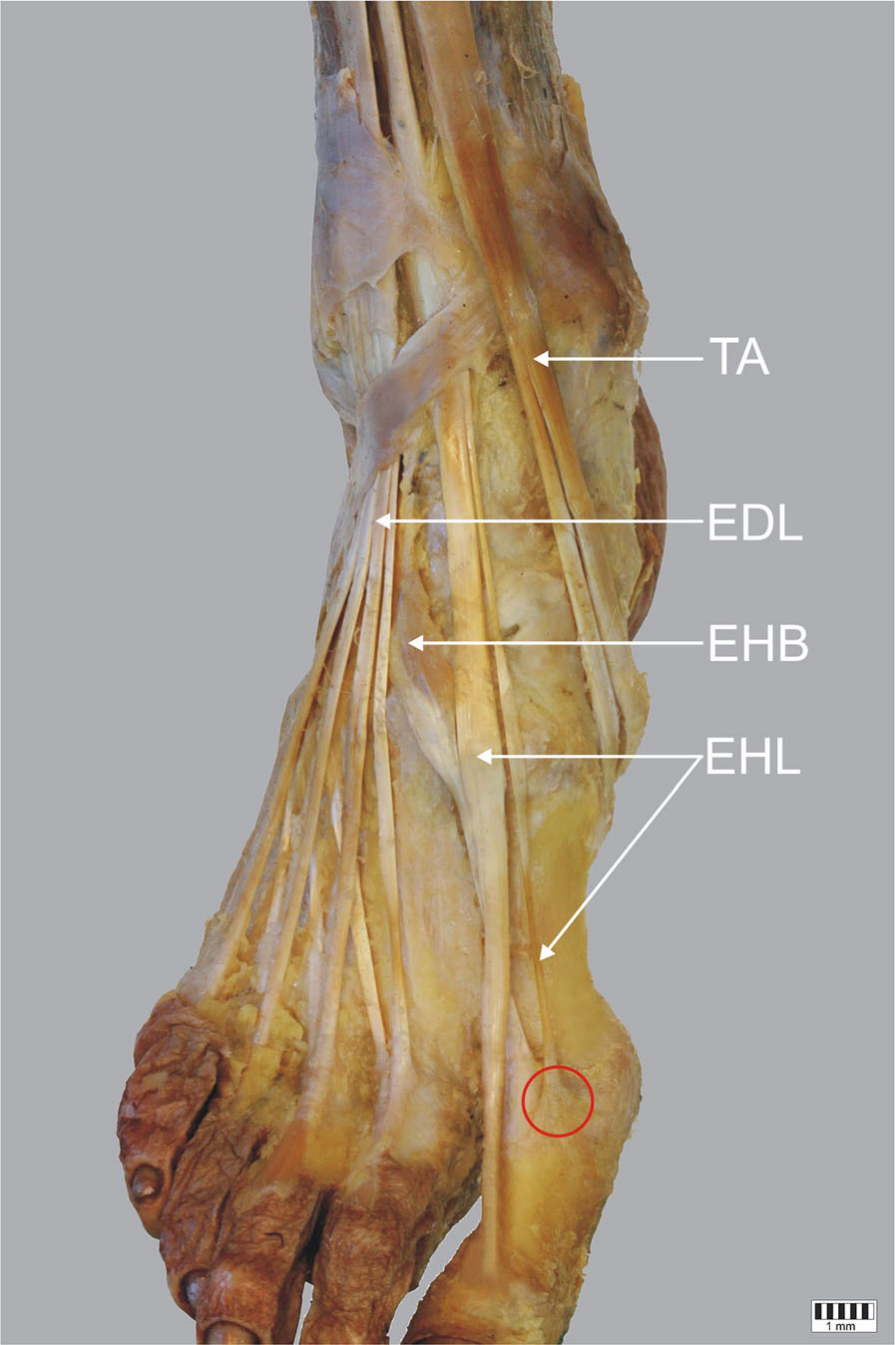
Type II a of insertion of the extensor hallucis longus tendon. Right lower limb. *EHL* extensor hallucis longus tendon *EHB* extensor hallucis brevis muscle *EDL* extensor digitorum longus *TA* tibialis anterior tendon. The red circle indicates the extension of the tendon

**Fig. 3:**
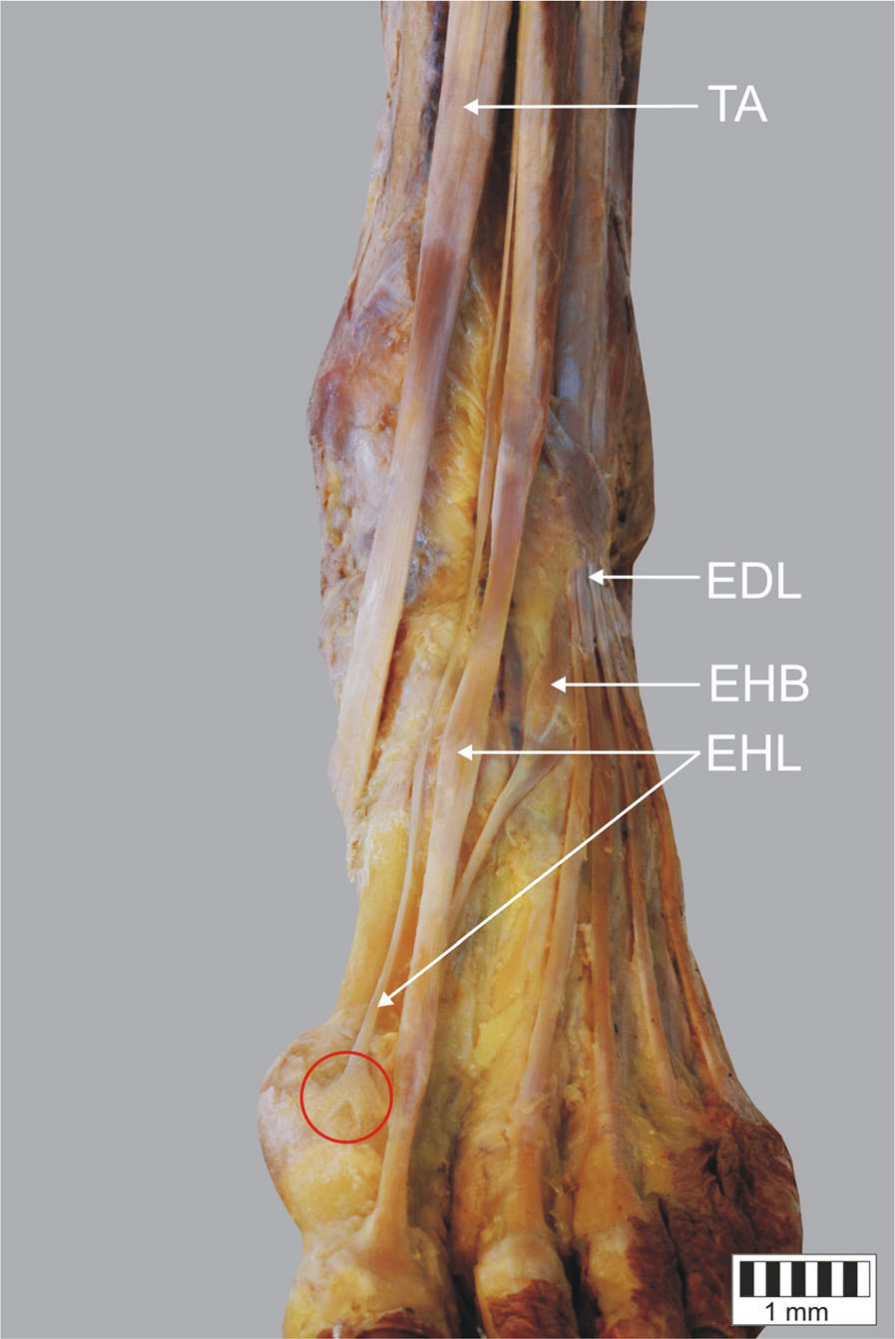
Type II b of insertion of the extensor hallucis longus tendon. Left lower limb. *EHL* extensor hallucis longus tendon *EHB* extensor hallucis brevis muscle *EDL* extensor digitorum longus *TA* tibialis anterior tendon. The red circle indicates the extension of the tendon

**Fig. 4:**
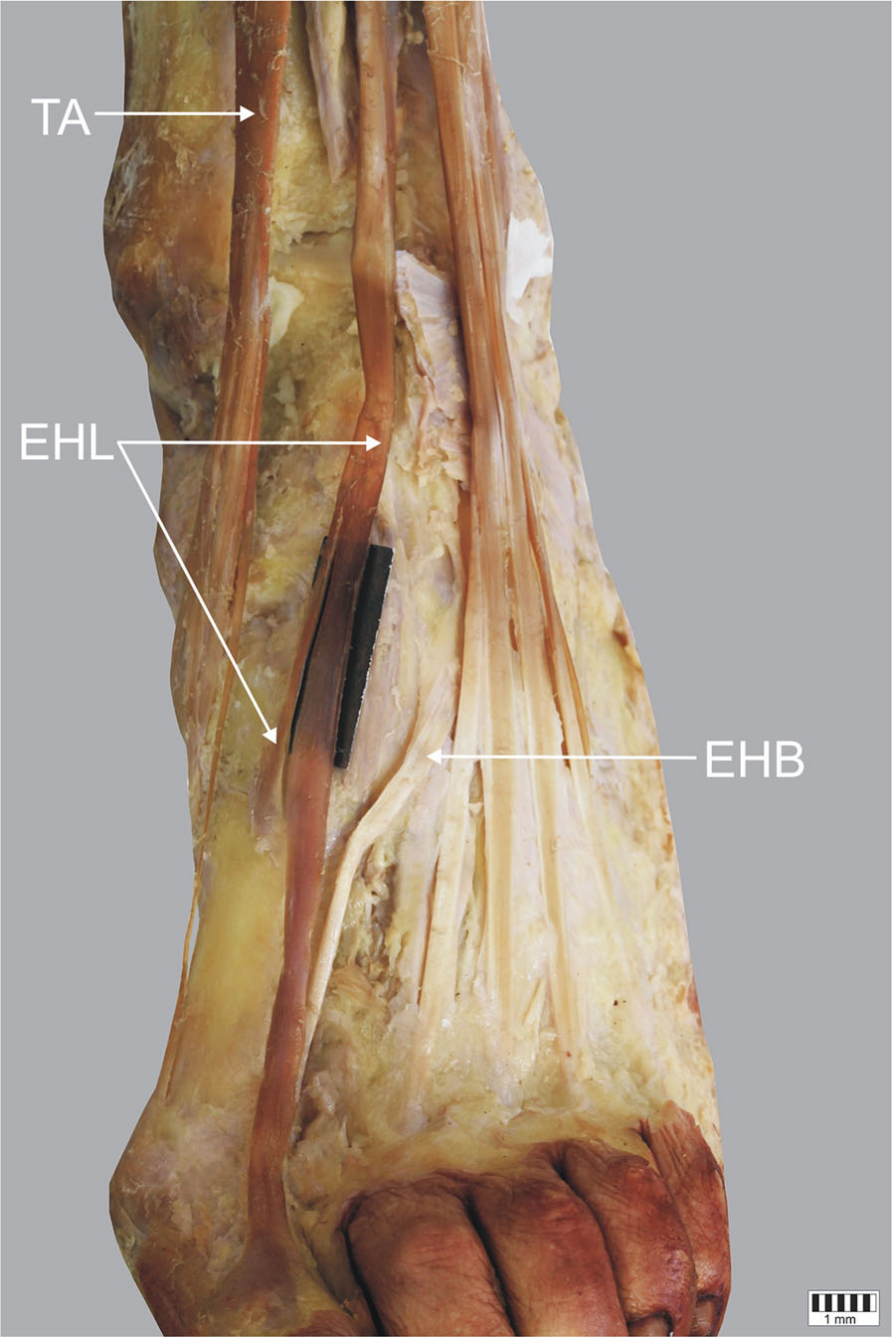
Type II **c** of insertion of the extensor hallucis longus tendon. Left lower limb. *EHL* extensor hallucis longus tendon *EHB* extensor hallucis brevis muscle *TA* tibialis anterior tendon

Type II – the EHL has two distal tendons. The dominant one ends as an extensor hood inserting to the distal phalanx of the big toe; however, three subtypes were distinguished according to the morphology of the auxiliary tendon:

- IIa – the auxiliary tendon inserts separately into the dorsal aspect of the proximal phalanx of the big toe, medial to the insertion of the extensor hallucis brevis tendon: present in 31 cases (29.9%) – Fig. 2.
- IIb – the auxiliary tendon inserts separately into the dorsal aspect of the proximal phalanx of the big toe, just distal to the to the insertion of the extensor hallucis brevis. The attachment is bifid: present in five cases (4.8%) – Fig. 3.
- IIc – the auxiliary tendon inserts into the dorsal aspect to the base of the first metatarsal: present in six cases (5.7%) – Fig. 4.

**Fig. 5:**
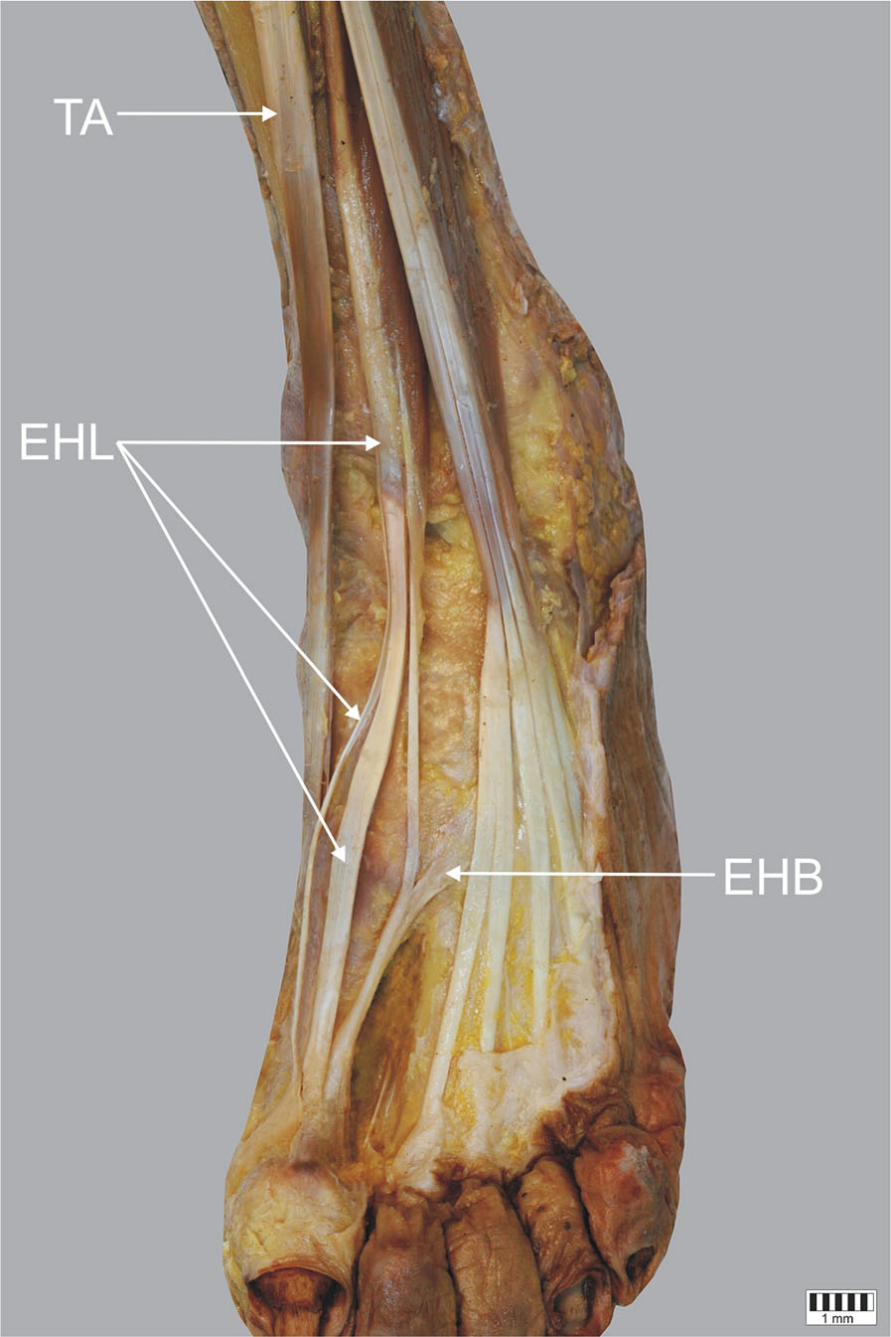
Type III of insertion of the extensor hallucis longus tendon. Left lower limb. *EHL* extensor hallucis longus tendon *EHB* extensor hallucis brevis muscle *TA* tibialis anterior tendon

**Fig. 6:**
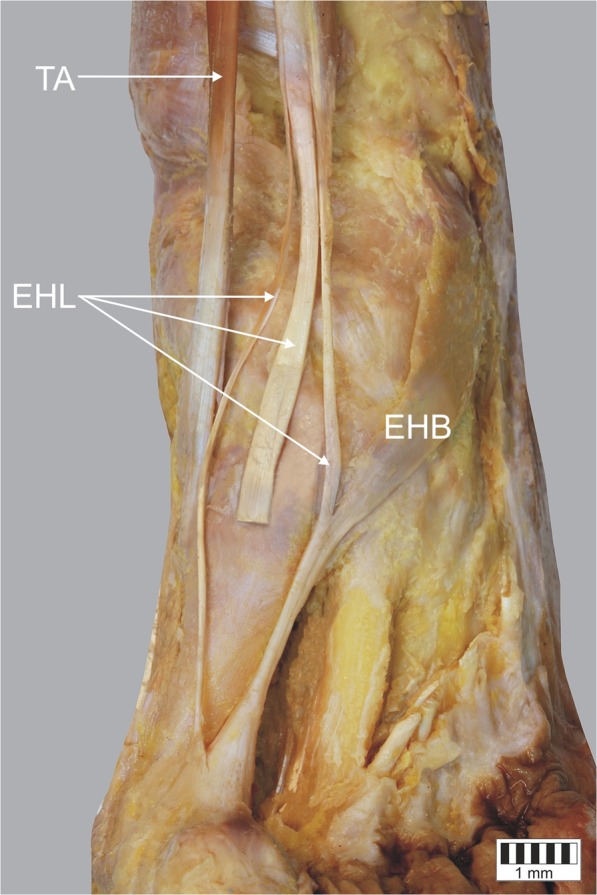
Type III of insertion of the extensor hallucis longus tendon. Left lower limb. *EHL* extensor hallucis longus tendon *EHB* extensor hallucis brevis muscle *TA* tibialis anterior tendon. Approximate view

Type III – the tendon splits into three bands: the main one inserts into the dorsal aspect of the distal part of the distal phalanx, a medial auxiliary band also inserts to the distal phalanx but more proximally, and a lateral auxiliary band (stronger of two auxiliary) fuses with the extensor halucis brevis and attaches to the proximal phalanx. This type was observed only in two cases (1.9%) – Figs. 5, 6.

There were significant differences in types distribution between genders (*p* = 0.00194) but not between body sides (*p* = 0.81348). Types IIa and IIc were recognised more often in males than in females: 23 vs. eight cases for IIa, and six vs. 0 cases for IIc. On the other hand, types IIb and III were more common in females than males: four vs. one case for IIb and two vs. 0 cases for III.

The morphometric parameters that differed significantly between the EHL types are presented in Table 1. Detailed data on differences in morphometric measurements according to gender, body side and EHL type are presented in the (Additional file 1: Table S1 and S2).

**Table 1:** Differences in morphometric measurements between types of EHL tendon

	Type I (*n* = 60) [mm]	Type II (*n* = 42) [mm]			Type III (*n* = 2) [mm]	*p*-value
		Subtype IIa (*n* = 31) [mm]	Subtype IIb (*n* = 5) [mm]	Subtype IIc (n = 6) [mm]		
Length of the leg	388.17 (36.66)*	389.94 (39.06)	402.20 (17.18)	430.00 (2.37)*	404.50 (55.86)	0.0680
Length of the EHL belly (from the origin to the tendon).	257.93 (29.89)	263.84 (27.74)	225.20 (36.81)*	283.50 (4.04)*	260.50 (54.45)	0.0398
Length of the EHL main tendon (from the origin to the insertion)	160.37 (26.00)†	165.73 (20.64)	198.62 (9.31)*†	151.97 (2.74)*	135.07 (31.32)	0.0053
EHL tendon width (origin)	4.22 (1.04)*	4.50 (0.91)	5.47 (0.72)*	4.09 (0.50)	3.68 (0.43)	0.0254
EHL tendon thickness (origin)	1.86 (0.58)*	2.17 (0.51) *†‡	1.42 (0.27)	1.65 (0.33)†	0.89 (0.04)‡	0.0003
Width of the EHL main tendon in ExP	5.88 (1.18)*	5.65 (1.28)*	5.49 (2.54)	3.69 (0.32)*	3.54 (0.04)	0.0009
Thickness of the EHL main tendon in ExP	1.82 (0.58)*	1.87 (0.77)*	1.58 (0.40)	1.09 (0.10)	1.33 (0.16)	0.0098
Distance between branching and attachment of the second band	–	3.83 (1.74)	–	–	7.97 (0.26)	0.0260

*, † and ‡ indicate groups that differ significantly between each other according to post-hoc test

## Discussion

The most important asset of this study is that it presents a new systematic classification of the EHL tendon based on anatomical dissectionSuch systematisation of existing classifications is needed to allow effective planning of surgical procedures.

A considerable degree of morphological variation can be attributed to phylogenetic development. Certain parts of the body, such as the palmaris longus or plantaris, can become reduced in response to evolutionary pressures [13–17], while others, such as the fibularis tetrius, can become more developed [4]. Hence, the presence of a high degree of variation in origin or insertion for a particular muscle may suggest that it has not yet completed its evolutionary development [4]; such a situation may observed for the EHL tendon, which was found to display a wide range of courses and insertion types, as well as additional bands [3–5, 18, 19]. However, the occurrence of such additional bands varies considerably from 80% [19], 81.25% (26 ft) [20] and 78.7% (34 ft) [18] to 35% (21 ft) [3], 26.5% (26 ft) [5] and 10% (six feet) [4]. In the present study, additional bands were found in 42.3% of cases (44 ft), this being the total number of types II and III.

More consistent results have been achieved regarding the course of the additional bands, the most frequently-observed variant being the medial course in relation to the main tendon, ranging from 93.4 to 100% of all cases [3, 5, 21]. This is confirmed in the present study, where the medial course of additional bands occurred in 98.1% (102 ft).

The first accurate classification was based on a study of 60 lower limbs and comprised three types of patterns (I-III) with subtypes [3]. We propose the addition of two further types/subtypes: Pattern II should be supplemented with Type II c identified in the present study, characterized by the auxiliary tendon inserting into the dorsal aspect of the base of the first metatarsal (six feet, 5.7%); in addition, Pattern III should be supplemented by our present Type III, characterized by a tendon split into three bands: a main tendon inserting into the dorsal aspect of the distal part of the distal phalanx, a medial auxiliary band inserting to the distal phalanx but more proximally, as well as a lateral auxiliary band (the stronger of the two auxiliary bands) fusing with the extensor halucis brevis and attaching to the proximal phalanx (two feet, 1.9%). A comparison of the Al-Saggaf classification with ours is presented in Table 2.

**Table 2:** Comparison results of the insertion of the extensor hallucis longus

Type	Al-Saggaf (2003)	Current study
Pattern I – single tendon, inserts on the dorsal aspect	39 (65%)	60 (57.5%)
Pattern II		
AT inserted into the dorsal aspect of the base of the proximal phalanx, just distal to the insertion of EHB.	9 (15%)	5 (4.8%)
AT joined to the termination part of the EHB and inserting into the dorsal aspect of the base of the proximal phalanx of the big toe.	3 (5%)	0
AT inserted into the dorsal aspect of the proximal phalanx of the big toe, medial to the insertion of the EHB.	2 (3.33%)	31 (29.9%)
AT joining the middle of EHB and forming a common tendon, and inserting into the dorsal aspect of the base of the proximal phalanx of the big toe.	2 (3.33%)	0
AT inserting into the dorsal aspect of the base of the first metatarsal.	0	6 (5.7%)
Pattern III		
Two ATs arising from the medial side of the main tendon and inserting into the capsule of the joint	3 (5%)	0
Two ATs arising from the medial and lateral sides of the main tendon and inserting into the capsule of the first metatarso-phalangeal joint	2 (3.33%)	0
Medial AT also inserts to the distal phalanx but more proximally, and lateral AT (the stronger of the two auxiliary bands) fuses with the extensor halucis brevis and attaches to the proximal phalanx	0	2 (1.9%)

Both Pattern II and Pattern III have an identical main tendon insertion as Pattern I. *AT* accessory tendon; *EHB* extensor hallucis brevis

Ankle extensor compartment trauma is commonly overlooked yet requires expeditious diagnosis and treatment in order to preserve function and provide positive outcomes. All components of the ankle extensor compartment are susceptible to injury, but the tendons are most frequently injured [22]. Of the different types of EHL lesions, one of the most typical is closed tendon ruptures, which are caused by active tendon contraction against resistance [23, 24]. Bone spurs or tight-fitting, high-laced boots which reduce one’s mechanical mobility can be predisposing factors; injuries resulting from sports-related activities such as ultramarathon running [25] and iatrogenic injuries after arthroscopic thermal ablation have also been described [26]. EHL tendon lacerations that have ocurred distal to the extensor expansion compartment may not significantly reduce function or tendon retraction and can thus be treated conservatively. Conversely, lacerations that are more proximal result in significant tendon retraction and reduced or lost function and require surgical intervention. The introduction of a new system of classification seems necessary because it is possible to speculate that additional bands prevent tendon laceration and retain normal muscle function at the site of the main tendon rupture.

Our study has some limitations. Its main weakness is that the sonographic and anatomic assessment methods were not applied on the same samples. Moreover, considering the heterogeneous nature of the anatomical area, the type of insertion or presence of accessory bands are the morphological details that our proposed classification is mostly focused on. Being within the limits of ability and realization of Ultrasound, MRI or biomechanics tests, these are the ones that should be used in further studies to develop this purpose. Offering a uniform system of classification and terminology we are willing to enlighten of “what and where” to look for in case of various injuries. Hopefully surgeons could also find this research useful for unified communication. Understanding these variants and implementation of the proposed classification system may be part of everyday specialists practice and could modify current surgical techniques. In addition, this work might serve as a segue into studies on diagnostic imaging to determine EHL morphological variations. Acceleration of future biomechanical and physiotherapeutic research could be possible due to our findings on EHL insertion types.

## Conclusion

The EHL tendon presents high morphological variability. The new classification introduces two new subtypes based on the course and insertion of the EHL tendon.

## Additional file

Additional file 1: **Table S1.** Differences in morphometric measurements between types of EHL. **Table S2.** Differences in morphometric measurements between genders and body sides. (DOCX 25 kb)

## Data Availability

Please contact authors for data requests (Łukasz Olewnik Ph. D - email address: lukasz.olewnik@umed.lodz.pl).

## Abbreviations

EDL: extensor digitorum longus
EHL: extensor hallucis longus
