# Supplementary material for: A cadaveric study of the morphology of the extensor hallucis longus - a proposal for a new classification
Source: BMC Musculoskelet Disord. 2019 Jul 3;20:310. doi: 10.1186/s12891-019-2688-8 (PMC6607556; doi:10.1186/s12891-019-2688-8)
Supplement: Supplementary file 1 — Table S1. Differences in morphometric measurements between types of EHL. Table S2. Differences in morphometric measurements between genders and body sides. (DOCX 25 kb) [file 12891_2019_2688_MOESM1_ESM.docx]

Additional file 1: Table S1 Differences in morphometric measurements between types of EHL.

|  | Type I (n=60) [mm] | Type II (n=42) [mm] | | | Type III (n=2) [mm] | p-value |
| --- | --- | --- | --- | --- | --- | --- |
|  |  | Subtype a (n=31) | Subtype b (n=5) | Subtype c (n=6) |  |  |
| Length of the crus | 388.17 (36.66)* | 389.94 (39.06) | 402.20 (17.18) | 430.00 (2.37)* | 404.50 (55.86) | 0.0680 |
| Length of the EHL belly | 257.93 (29.89) | 263.84 (27.74) | 225.20 (36.81)* | 283.50 (4.04)* | 260.50 (54.45) | 0.0398 |
| Length of the EHL main tendon | 160.37 (26.00)† | 165.73 (20.64) | 198.62 (9.31)*† | 151.97 (2.74)* | 135.07 (31.32) | 0.0053 |
| EHL tendon width (origin) | 4.22 (1.04)* | 4.50 (0.91) | 5.47 (0.72)* | 4.09 (0.50) | 3.68(0.43) | 0.0254 |
| EHL tendon thickness (origin) | 1.86 (0.58)* | 2.17 (0.51)*†‡ | 1.42 (0.27) | 1.65 (0.33)† | 0.89 (0.04)‡ | 0.0003 |
| Distances to the origin of the I band | - | 29.91 (22.13) | 56.82 (5.36) | 68.41 (3.96) | 8.33 (2.18) | 1.0000 |
| Width of the EHL main tendon in ExP | 5.88 (1.18)* | 5.65 (1.28)* | 5.49 (2.54) | 3.69 (0.32)* | 3.54 (0.04) | 0.0009 |
| Thickness of the EHL main tendon in ExP | 1.82 (0.58)* | 1.87 (0.77)* | 1.58 (0.40) | 1.09 (0.10) | 1.33 (0.16) | 0.0098 |
| ExP – distance from attachment to PK | 6.02 (1.95) | 22.83 (94.51) | 8.34 (0.67) | 3.32 (0.98) | - | 1.0000 |
| Length of the EHL II band | - | 95.22 (25.94) | 155.64 (9.00) | 65.82 (4.66) | 98.78 (3.60) | 1.0000 |
| Length of the EHL medial band | - | - | 4.25 (0.98) | - | - | - |
| Length of the EHL lateral band | - | - | 4.41 (2.36) | - | - | - |
| Width of the EHL II band | - | 1.64 (0.78) | 1.71 (0.34) | 2.79 (0.49) | 0.75(0.04) | 1.0000 |
| Thickness of the EHL II band | - | 0.80 (0.33) | 0.54 (0.21) | 1.05 (0.21) | 0.72 (0.14) | 1.0000 |
| Width of the ExP medial band | - | - | 6.19 (0.53) | - | - | - |
| Width of the ExP lateral band | - | - | 3.73 (0.49) | - | - | - |
| Width of the II band at ExP | - | 3.31 (0.99) | - | - | 2.94 (0.09) | 0.6506 |
| Thickness of the II band at ExP | - | 0.83 (0.43) | - | - | 1.18 (0.04) | 0.2129 |
| Distance between branching and attachment of the II band | - | 3.83 (1.74) | - | - | 7.97 (0.26) | 0.0260 |
| Length of the EHL III band | - | - | - | - | 77.70 (2.11) | 1.0000 |
| Width of the EHL III band (origin) | - | - | - | - | 1.21 (0.04) | 1.0000 |
| Thickness of the EHL III band (origin) | - | - | - | - | 0.64 (0.05) | 1.0000 |
| Width of the III band at ExP | - | - | - | - | 2.22 (0.14) | 1.0000 |
| Thickness of the III band at ExP | - | - | - | - | 0.47 (0.05) | 1.0000 |
| Distance between branching and attachment of the III band | - | - | - | - | 101.00 (0.00) | 1.0000 |

Additional file 1: Table S2. Differences in morphometric measurements between genders and body sides.

|  | Females (n=42)  [mm] | Males (n=62)  [mm] | p-value | Right (n=51) [mm] | Left (n=53) [mm] | p-value |
| --- | --- | --- | --- | --- | --- | --- |
| Length of the crus | 399.77 (39.34) | 380.76 (29.82) | 0.0083 | 393.29 (36.01) | 390.94 (37.95) | 0.7848 |
| Length of the EHL belly | 267.61 (27.87) | 247.88 (30.12) | 0.0022 | 260.57 (31.18) | 258.75 (29.62) | 0.7182 |
| Length of the EHL main tendon | 165.00 (18.44) | 159.64 (31.95) | 0.1450 | 162.19 (22.85) | 163.46 (26.73) | 0.8862 |
| EHL tendon width (origin) | 4.59 (1.01) | 4.00 (0.86) | 0.0097 | 4.36 (0.93) | 4.34 (1.06) | 0.7574 |
| EHL tendon thickness (origin) | 2.05 (0.56) | 1.68 (0.54) | 0.0007 | 1.93 (0.59) | 1.88 (0.57) | 0.7451 |
| Distances to the origin of the I band | 33.99 (24.38) | 44.18 (24.42) | 0.2464 | 38.13 (24.67) | 36.17 (25.07) | 0.7863 |
| Width of the EHL main tendon in ExP | 5.73 (1.42) | 5.45 (1.29) | 0.5378 | 5.67 (1.28) | 5.57 (1.46) | 0.5606 |
| Thickness of the EHL main tendon in ExP | 1.76 (0.72) | 1.80 (0.51) | 0.6692 | 1.89 (0.72) | 1.66 (0.54) | 0.1249 |
| ExP – distance from insertion to PK | 14.51 (67.39) | 5.99 (1.79) | 0.9695 | 6.06 (1.86) | 15.92 (73.00) | 0.6392 |
| Length of the EHL II band | 95.29 (31.83) | 104.56 (32.23) | 0.2413 | 99.37 (31.54) | 96.87 (33.04) | 0.7325 |
| Length of the EHL medial band | 5.99 (0.00) | 3.82 (0.15) | 1.0000 | 3.77 (0.16) | 4.58 (1.23) | 0.3865 |
| Length of the EHL lateral band | 8.63 (0.00) | 3.36 (0.12) | 1.0000 | 3.27 (0.08) | 5.18 (2.99) | 0.1489 |
| Width of the EHL II band | 1.93 (0.91) | 1.42 (0.41) | 0.0696 | 1.78 (0.81) | 1.75 (0.85) | 0.8690 |
| Thickness of the EHL II band | 0.86 (0.32) | 0.68 (0.30) | 0.0890 | 0.83 (0.37) | 0.76 (0.26) | 0.4650 |
| Width of the ExP medial band | 7.11 (0.00) | 5.96 (0.16) | 1.0000 | 5.83 (0.09) | 6.43 (0.59) | 0.1489 |
| Width of the ExP lateral band | 3.93 (0.00) | 3.68 (0.56) | 1.0000 | 4.16 (0.07) | 3.44 (0.42) | 0.1489 |
| Width of the II band at ExP | 3.42 (0.98) | 2.98 (0.90) | 0.1961 | 3.33 (0.95) | 3.23 (1.02) | 0.7021 |
| Thickness of the II band at ExP | 0.86 (0.43) | 0.84 (0.42) | 0.8601 | 0.93 (0.32) | 0.74 (0.52) | 0.1216 |
| Distance between branching and attachment of the II band | 3.83 (1.70) | 4.65 (2.47) | 0.5437 | 4.06 (2.08) | 4.10 (1.85) | 0.6489 |
| Length of the EHL III band | - | 77.70 (2.11) | 1.0000 | 76.21 (0.00) | 79.19 (0.00) | 1.0000 |
| Width of the EHL III band (origin) | - | 1.21 (0.04) | 1.0000 | 1.23 (0.00) | 1.18 (0.00) | 1.0000 |
| Thickness of the EHL III band (origin) | - | 0.64 (0.05) | 1.0000 | 0.67 (0.00) | 0.60 (0.00) | 1.0000 |
| Width of the III band at ExP | - | 2.22 (0.14) | 1.0000 | 2.32 (0.00) | 2.12 (0.00) | 1.0000 |
| Thickness of the III band at ExP | - | 0.47 (0.05) | 1.0000 | 0.43 (0.00) | 0.50 (0.00) | 1.0000 |
| Distance between branching and attachment of the III band | - | 101.00 (0.00) | 1.0000 | 101.00 (0.00) | 101.00 (0.00) | 1.0000 |
